# Synthesis and Biological Evaluation of New 2-Azetidinones with Sulfonamide Structures

**DOI:** 10.3390/molecules18044140

**Published:** 2013-04-08

**Authors:** Oana Maria Dragostin, Florentina Lupascu, Cornelia Vasile, Mihai Mares, Valentin Nastasa, Ramona Florina Moraru, Dragos Pieptu, Lenuta Profire

**Affiliations:** 1 Department of Pharmaceutical Chemistry, Faculty of Pharmacy, Grigore T. Popa University of Medicine and Pharmacy, 16 University Street, Iasi 700115, Romania; E-Mails: farmacist_oanamaria@yahoo.com (O.M.D.); geani0407@yahoo.com (F.L.); 2 Department of Physical Chemistry of Polymers, Petru Poni Institute of Macromolecular Chemistry, Romanian Academy, 41A Grigore Ghica Voda Alley, Iasi 700487, Romania; E-Mail: cvasile@icmpp.ro; 3 Laboratory of Antimicrobial Chemotherapy, Faculty of Veterinary Medicine, Ion Ionescu de la Brad University of Agricultural Sciences and Veterinary Medicine, 8 Mihail Sadoveanu Alley, Iasi 700489, Romania; E-Mails: mycomedica@gmail.com (M.M.); alvali2003@yahoo.com (V.N.); moraru82@gmail.com (R.F.M.); 4 Department of Plastic Surgery, Faculty of Medicine, Grigore T. Popa University of Medicine and Pharmacy, 16 University Street, Iasi 700115, Romania

**Keywords:** sulfonamide, azetidinone, synthesis, antimicrobial activity, antioxidant effect

## Abstract

New series of *N*-(arylidene)hydrazinoacetyl sulfonamides ****4a**_1_**_–**6**_, ****4b**_1_**_–**6**_ and *N*-(4-aryl-3-chloro-2-oxoazetidin-1-yl)aminoacetyl sulfonamides ****5a**_1_**_–**6**_, ****5b**_1_**_–**6**_ were synthesized. The structures of the new derivatives was confirmed using spectral methods (FT-IR, ^1^H-NMR, ^13^C-NMR). The antibacterial activities of these compounds against Gram positive (*Staphyloccoccus aureus* ATCC 6583, *Staphyloccoccus epidermidis* ATCC 12228, *Enterococcus faecalis* ATCC 25912) and Gram negative (*Klebsiella pneumoniae* CIP 53153, *Proteus vulgaris* CIP 104989, *Citrobacter freundii* CIP 5732, *Enterobacter cloacae* CIP 103475, *Escherichia coli* ATCC 25922, *Pseudomonas aeruginosa* CIP 82118) bacterial strains were evaluated using the broth micro-dilution method. Compound ****4a**_2_** displayed the highest antibacterial activity, especially against *Staphyloccoccus epidermidis*, *Enterococcus faecalis* and *Pseudomonas aeruginosa*. The antioxidant potential of the synthesized compounds was also investigated according to ferric reducing power, total antioxidant activity and DPPH radical scavenging assays. All tested compounds showed excellent antioxidant activity in comparison with sulfadiazine and sulfisoxazole which were used as parent sulfonamides. Moreover, some of them showed an antioxidant activity comparable with that of ascorbic acid. In general, the compounds designed based on a sulfadiazine skeleton (compounds ****4a**_1–6_**, ****5a**_1–6_**) are more active than those obtained from sulfisoxazole (compounds ****4b**_1–6_**, ****5b**_1–6_**), and the *N*-(arylidene)hydrazinoacetyl sulfonamide derivatives ****4a**_1_**_–**6**_, ****4b**_1_**_–**6**_ are more active than their azetidionone analogues ****5a**_1_**_–**6**_, ****5b**_1_**_–**6**_.

## 1. Introduction

The 2-azetidinone skeleton, otherwise known as the β-lactam ring, has been recognized as a useful building block in the synthesis of biologically important compounds. Azetindin-2-one derivatives display interesting biological activities such as antifungal, antimicrobial [1,2,3,4], antitubercular [5,6], analgesic, anti-inflammatory [7,8], chymase inhibitory [9], antitumoral [10,11,12], antiviral, antidiabetic and cholesterol absorption inhibitory properties [13]. The activity of famous antibiotic classes such as the penicillins, cephalosporins, carumonam, aztreonam, thienamicine, nocardicins and carbapenems is attributed to the presence of an 2-azetidinone ring [2]. Unfortunately, the most widely used of them exert selective pressure on bacteria and permit the proliferation of resistant organisms. Several synthetic and semi-synthetic β-lactam antibiotics were developed due to the growing resistance of bacteria towards the classical β-lactam antibiotics and the need for drugs with a more specific antibacterial activity [1]. The biological activity of sulfonamides is also well documented. They have be found to be useful in a variety of applications, including antibacterial, antifungal, antitumor agents, diuretics, carbonic anhydrase inhibitors, hypoglycemic agents, thyroid inhibitors, anticonvulsants and protease inhibitors [14,15]. Among antibacterial sulfonamides, sulfadiazine and its silver and cerium salts have an important place. They are widely used as topical agents for the management of burns where they prevent infections and promote rapid healing with minimal scarring [15].

Wounds are physical injuries that result in an opening of the skin. Proper healing of wounds is essential for the restoration of disrupted anatomical continuity and disturbed functional status of the skin [16]. Normal healing of wounds is a dynamic process following three phases: inflammation, granulation (tissue formation) and re-epithelization (tissue remodeling), which overlap in time [17]. It was proven that reactive oxygen species (ROS) and bacterial infections are deleterious to the wound healing process due to their harmful effects on cells and tissues [18]. ROS are produced in high amounts at wound sites as a defense mechanism against invading bacteria. At the same time, the process of wound healing may be hampered by the presence of free radicals, which can damage the cells surrounding the wound, or by microbial infection [19] and recent data has proved the beneficial effects of antioxidants in the wound healing process [20,21,22]. In the present study, we are reporting the design, synthesis and biological evaluation of some new 2-azetidinone derivatives of sulfadiazine and sulfisoxazole with potential use in wound healing processes.

## 2. Results and Discussion

### 2.1. Chemistry

**Scheme 1:**
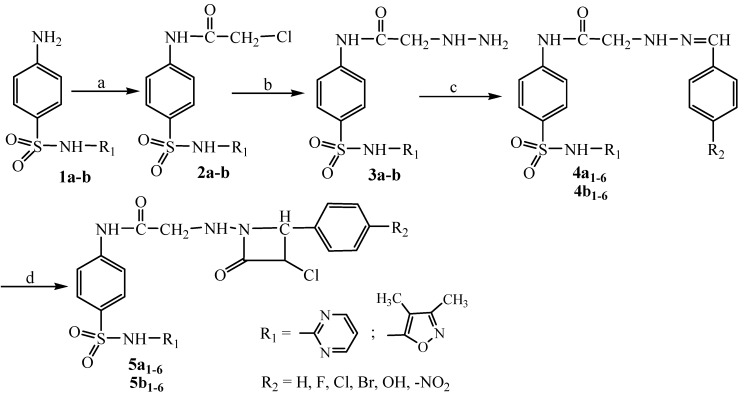
Synthesis of azetidinone derivatives (**5a**_1–6_, **5b**_1–6_).

Azetidinone derivatives **5a**_1–6_, **5b**_1–6_ were prepared using the method summarized in Scheme 1. First, sulfadiazine (4-amino-*N*-pyrimidin-2-yl-benzensulfonamide, **1a**) and sulfisoxazole [4-amino-*N*-(3,4-dimethyl-1,2-oxazol-5-yl)benzensulfonamide, **1b**] were reacted with chloroacetyl chloride whereby the corresponding chloracetyl derivatives **2a**–**b** were obtained. Compounds **2a**–**b** on amination with hydrazine hydrate afforded hydrazinoacetyl sulfonamide derivatives **3a**–**b** [23]. The condensation reaction of compounds **3a**–**b** with various aromatic aldehydes yielded *N*-(arylidene)hydrazinoacetyl sulfonamide derivatives **4a**_1–6_, **4b**_1–6_. Finally, the compounds **4a**_1–6_, **4b**_1–6_ upon reaction with chloracetyl chloride in the presence of triethylamine afforded *N*-(4-aryl-3-chloro-2-oxoazetidin-1-yl)aminoacetyl sulfonamides **5a**_1–6_, **5b**_1–6_.

*Reagents and Conditions:* (**a**) chloracetyl chloride, dry acetone, anhydrous K_2_CO_3,_ heating 12 h; (**b**) hydrazine hydrate 99%, ethanol, heating 10 h; (**c**) aromatic aldehydes, acetic acid, ethanol 50%, heating 8 h; (**d**) chloracetyl chloride, anhydrous 1,4-dioxane, triethylamine, room temperature, stirrer 3 h.

The structure of the compounds was assigned on the basis of spectral (IR, ^1^H-NMR, ^13^C-NMR) data. The IR spectra of compounds **4a**_1–6_ (sulfadiazine series) showed absorption bands for the -CH_2_-NH- group in the range of 2830–2853 cm^−1^, for the NH-CO group in the range of 1622–1623 cm^−1^ and for the characteristic azomethine group (CH=N) in the 1534–1539 cm^−1^ range. In the spectra of the sulfisoxazole derivatives **4b**_1–6_ the characteristic absorption bands were observed in the region of 2842–2870 cm^−1^ (-CH_2_-NH-), 1622–1629 cm^−1^ (NH-CO) and 1507–1540 cm^−1^ (CH=N). In the ^1^H-NMR spectra of the *N*-(arylidene)hydrazinoacetyl sulfonamides **4a**_1–6_, **4b**_1–6_ the -CH_2_-NH methylene protons resonated as a doublet in the 3.56–3.79 ppm region, while the proton of the azomethine group (N=CH) appeared as a singlet in the 8.06–8.22 ppm region. In the IR spectra of the azetidinone derivatives **5a**_1–6_, **5b**_1–6_ the carbonyl group of the β-lactam ring appeared as a characteristic absorption band in the range of 1739–1745 cm^−1^ and 1739–1752 cm^−1^, respectively. The IR absorption bands and ^1^H-NMR signals characteristic of the azomethine group disappeared from the spectra of the azetidinone derivatives, which confirms that the cyclization reaction with chloracetyl chloride took place. The ^1^H-NMR spectra of **5a**_1–6_ (sulfadiazine series) and **5b**_1–6_ (sulfisoxazole series) showed two doublets, which are characteristic for N-CH and CH-Cl that appear in the range of 5.32–5.45 and 5.02–5.23 ppm, respectively. In ^13^C-NMR spectra of the azetidinone derivatives, the characteristic signals for a β-lactam ring (CH-NH, CH-Cl, CO cyclic) appeared in the range of 67.8–76.1, 61.04–64.3 and 160.3–162.8 ppm (**5a**_1–6_ series) and 75.9–79.1, 64.2–67.0 and 160.3–168.2 ppm (**5b**_1–6_ series) respectively. The spectral data lend strong support to the proposed structures of all the synthesized compounds.

### 2.2. Biological Evaluation

#### 2.2.1. Antibacterial Activity

The antibacterial activities of the title compounds were evaluated using the broth micro-dilution method [24] and the results are listed Table 1 and Table 2.

**Table 1:** MIC values (µg/mL) of the sulfadiazine derivatives **4a**_1–6_, **5a**_1–6_.

Sample	MICs * values (µg/mL)								
	SA	SE	EF	KP	PV	CF	EC ^a^	EC ^b^	PA
**4a**_1_	>512	>512	>512	>512	>512	>512	>512	>512	256
**4a**_2_	>512	128	256	>512	>512	>512	>512	>512	128
**4a**_3_	>512	>512	>512	>512	>512	>512	>512	>512	>512
**4a**_4_	>512	>512	>512	>512	>512	>512	>512	>512	128
**4a**_5_	>512	>512	>512	>512	>512	>512	>512	>512	>512
**4a**_6_	>512	>512	>512	>512	>512	>512	>512	>512	>512
**5a**_1_	>512	>512	>512	>512	>512	>512	>512	>512	>512
**5a**_2_	>512	>512	>512	>512	>512	>512	>512	>512	>512
**5a**_3_	>512	>512	>512	>512	>512	>512	>512	>512	>512
**5a**_4_	>512	>512	>512	>512	>512	>512	>512	>512	>512
**5a**_5_	>512	>512	>512	>512	>512	>512	>512	>512	>512
**5a**_6_	>512	>512	>512	>512	>512	>512	>512	>512	>512
**S**	>1000	>1000	>1000	>1000	>1000	>1000	>1000	>1000	800
**A**	35.8	3	3	16	-	-	8	35.8	128

* Mean values (n = 3); SA: *Staphyloccoccus aureus* ATCC 6583; SE: *Staphyloccoccus epidermidis* ATCC 12228; EF: *Enterococcus faecalis* ATCC 25912; KP: *Klebsiella pneumoniae* CIP 53153; PV: *Proteus vulgaris* CIP 104989; CF: *Citrobacter freundii* CIP 5732; EC ^a^: *Enterobacter cloacae* CIP 103475; EC ^b^: *Escherichia coli* ATCC 25922; PA: *Pseudomonas aeruginosa* CIP 82118; S: Sulfanilamide; A: Ampicillin.

**Table 2:** MIC values (µg/mL) of the sulfisoxazole derivatives **4b**_1–6_, **5b**_1–6_.

Sample	MICs * values (µg/mL)								
	SA	SE	EF	KP	PV	CF	EC ^a^	EC ^b^	PA
**4b**_1_	>512	>512	>512	>512	>512	>512	>512	>512	>512
**4b**_2_	>512	>512	>512	>512	>512	>512	>512	>512	>512
**4b**_3_	>512	>512	>512	>512	>512	>512	>512	>512	>512
**4b**_4_	>512	>512	>512	>512	>512	>512	>512	>512	>512
**4b**_5_	>512	>512	>512	>512	>512	>512	>512	>512	256
**4b**_6_	>512	>512	>512	>512	>512	>512	>512	>512	>512
**5b**_1_	>512	>512	>512	>512	>512	>512	>512	>512	>512
**5b**_2_	>512	>512	>512	>512	>512	>512	>512	>512	>512
**5b**_3_	>512	>512	>512	>512	>512	>512	>512	>512	>512
**5b**_4_	>512	>512	>512	>512	>512	>512	>512	>512	>512
**5b**_5_	>512	>512	>512	>512	>512	>512	>512	>512	>512
**5b**_6_	>512	>512	>512	>512	>512	>512	>512	>512	>512
**S**	>1000	>1000	>1000	>1000	>1000	>1000	>1000	>1000	800
**A**	35.8	3	3	16	-	-	8	35.8	128

* Mean values (n = 3); SA: *Staphyloccoccus aureus* ATCC 6583; SE: *Staphyloccoccus epidermidis* ATCC 12228; EF: *Enterococcus faecalis* ATCC 25912; KP: *Klebsiella pneumoniae* CIP 53153; PV: *Proteus vulgaris* CIP 104989; CF: *Citrobacter freundii* CIP 5732; EC ^a^: *Enterobacter cloacae* CIP 103475; EC ^b^: *Escherichia coli* ATCC 25922; PA: *Pseudomonas aeruginosa* CIP 82118; S: Sulfanilamide; A: Ampicillin.

The minimum inhibitory concentrations (MICs) of almost all compounds were more than 512 μg/mL. The *N*-(arylidene)hydrazinoacetyl derivative of sulfadiazine (compound **4a**_2_) was the most active compound, as it was active on *Staphyloccoccus epidermidis* ATCC 12228 (128 μg/mL), *Enterococcus faecalis* ATCC 25912 (256 μg/mL) and *Pseudomonas aeruginosa* CIP 82118 (128 μg/mL). The compounds **4a**_1_ (256 μg/mL), **4a**_4_ (128 μg/mL) and **4b**_5_ (256 μg/mL) were active against *Pseudomonas aeruginosa*. All tested compounds are more active than sulfanilamide, but less active than ampicillin used as positive controls.

#### 2.2.2. Antioxidant Activity

#### 2.2.2.1. Ferric Reducing Power

The measurement of reducing power defines an important aspect of the antioxidant activity of the compounds. In this assay, the presence of a reducing agent in the sample results in reducing of the ferric/ferricyanide complex to its ferrous (Fe^2+^) form. The amount of Fe^2+^ is then quantitatively monitored by measuring the intensity of Perl’s Prussian blue colour complex at 695 nm [25]. The results expressed as EC_50_ values (mg/mL) are presented in Table 3 and Table 4. The small value of the EC_50_ indicates a higher ferric reducing power.

**Table 3:** Ferric reducing power (EC_50_ mg/mL) of the sulfadiazine derivatives **4a**_1–6_, **5a**_1–6_.

Sample	EC_50_ mg/mL	Sample	EC_50_ mg/mL
**4a**_1_	0.0663 ± 0.0056	**5a**_1_	0.1553 ± 0.0152
**4a**_2_	0.0756 ± 0.0040	**5a**_2_	0.1376 ± 0.0002
**4a**_3_	0.0856 ± 0.0051	**5a**_3_	0.1745 ± 0.0125
**4a**_4_	0.0790 ± 0.0026	**5a**_4_	0.1798 ± 0.0018
**4a**_5_	0.0510 ± 0.0036	**5a**_5_	0.0945 ± 0.0085
**4a**_6_	0.0503 ± 0.0025	**5a**_6_	0.2277 ± 0.0037
**1a**	2.6140 ± 0.0301	**AA**	0.0075 ± 0.0002

Data are mean ± SD (n = 3, *p* < 0.05).

As it can be seen both *N*-(arylidene)hydrazinoacetyl and azetidinone derivatives are more active than their sulfonamide parents, sulfadiazine (**1a**) and sulfizoxazole (**1b**). In the *N*-(arylidene) hydrazinoacetyl series of sulfadiazine (compounds **4a**_1__–__6_) it was observed that the most active compounds were those which resulted from reaction of condensation with 4-hydroxybenzaldehyde (compound **4a**_5_) and 4-nitrobenzaldehyde (compound **4a**_6_). The values of EC_50_ for these compounds were 0.0510 ± 0.0036 (compound **4a**_5_) and 0.0503 ± 0.0025 (compound **4a**_6_), which means that they are about 50 times more active than sulfadiazine (EC_50_ = 2.6140 ± 0.0301). Concerning the azetidinone series the most active compound was **5a**_5_, which is the analogue of **4a**_5_ in the azetidinone series. This compound is approximately 28 time more active (EC_50_ = 0.0945 ± 0.0085) then sulfadiazine (EC_50_ = 2.6140 ± 0.0301) (Table 3). The ferric reducing power of the compounds resulting through modulation of sulfisoxazole is lower than that of the the analogues of the sulfadiazine series. In reference to sulfisoxazole (1b), all tested compounds **4b**_1–6_, **5b**_1–6_ are more active. The most active compounds were **4b**_5_ [*N*-(arylidene)hydrazinoacetyl series] and **5b**_5_ (azetidinone series), which have in their structure the 4-hydroxyphenyl radical. These compounds are 46 times (**4b**_5_, EC_50_ = 0.0210 ± 0.0065) and 10 times (**5b**_5_, EC_50_ = 0.0935 ± 0.0098) more active, respectively, than sulfisoxazole (1b, EC_50_ = 0.9640 ± 0.0443) (Table 4). 

**Table 4:** Ferric reducing power (EC_50_ mg/mL) of the sulfisoxazole derivatives **4b**_1–6_, **5b**_1–6_.

Sample	EC_50_ mg/mL	Sample	EC_50_ mg/mL
**4b**_1_	0.0756 ± 0.0055	**5b**_1_	0.1450 ± 0.0003
**4b**_2_	0.2043 ± 0.0055	**5b**_2_	0.1164 ± 0.0025
**4b**_3_	0.0610 ± 0.0036	**5b**_3_	0.1915 ± 0.0216
**4b**_4_	0.0612 ± 0.0040	**5b**_4_	0.3182 ± 0.0411
**4b**_5_	0.0210 ± 0.0065	**5b**_5_	0.0935 ± 0.0098
**4b**_6_	0.1173 ± 0.0066	**5b**_6_	0.1106 ± 0.0149
**1b**	0.9640 ± 0.0443	**AA**	0.0075 ± 0.0002

Data are mean ± SD (n = 3, *p* < 0.05).

The chemical modulation of the parent sulfonamides improve their ferric reducing power and all tested compounds are more active than sulfadiazine and sulfisoxazole, respectively, but they are less active than ascorbic acid (AA) at the same concentration.

#### 2.2.2.2. Total Antioxidant Activity

The total antioxidant activity was determined using phophomolybdenum blue complex with a maximum absorption at 695 nm [26]. The data presented in Table 5 and Table 6 show that the tested compounds are more active than sulfadiazine and sulfisoxazole, respectively, and moreover, the sulfadiazine derivatives are more active than sulfisoxazole compounds. 

**Table 5:** Total antioxidant activity (EC_50_ mg/mL) of the sulfadiazine derivatives **4a**_1–6_, **5a**_1–6_.

Sample	EC_50_ mg/mL	Sample	EC_50_ mg/mL
**4a**_1_	0.0180 ± 0.0044	**5a**_1_	0.0398 ± 0.0022
**4a**_2_	0.0280 ± 0.0067	**5a**_2_	0.0498 ± 0.0015
**4a**_3_	0.0110 ± 0.0007	**5a**_3_	0.0330 ± 0.0098
**4a**_4_	0.0360 ± 0.0089	**5a**_4_	0.0507 ± 0.0037
**4a**_5_	0.0440 ± 0.0050	**5a**_5_	0.0563 ± 0.0009
**4a**_6_	0.0220 ± 0.0072	**5a**_6_	0.0341 ± 0.0055
**1a**	6.6483 ± 0.0180	**AA**	0.0067 ± 0.0003

Data are mean ± SD (n = 3, *p* < 0.05).

**Table 6:** Total antioxidant activity (EC_50_ mg/mL) of the sulfisoxazole derivatives **4b**_1–6_, **5b**_1–6_.

Sample	EC50 mg/mL	Sample	EC50 mg/mL
**4b**_1_	0.0481 ± 0.0042	**5b**_1_	0.0433 ± 0.0009
**4b**_2_	0.0612 ± 0.0078	**5b**_2_	0.0756 ± 0.0033
**4b**_3_	0.0330 ± 0.0009	**5b**_3_	0.0574 ± 0.0025
**4b**_4_	0.0332 ± 0.0047	**5b**_4_	0.0385 ± 0.0078
**4b**_5_	0.0551 ± 0.0086	**5b**_5_	0.0718 ± 0.0008
**4b**_6_	0.0794 ± 0.0091	**5b**_6_	0.0825 ± 0.0045
**1b**	21.658 ± 0.0224	**AA**	0.0067 ± 0.0003

Data are mean ± SD (n = 3, *p* < 0.05).

The most favorable influence seems to be the presence of halogen on the phenyl ring, especially the presence of chlorine in the sulfadiazine series (compounds **4a**_3_, **5a**_3_) and the presence of chlorine and bromine in the sulfisoxazole series (compounds **4b**_3–4_, **5b**_3–4_). The compound **4a**_3_ (EC_50_ = 0.0110 ± 0.0007) is about 600 times more active than sulfadiazine (**1a**) (EC_50_ = 6.6483 ± 0.0180) and its antioxidant activity is comparable with the activity of ascorbic acid (**AA**) (EC_50_ = 0.0067 ± 0.0003). Although its azetidinone analogue **5a**_3_ has a lower activity, it remains significant in reference with sulfadiazine (Table 5). In the sulfisoxazole series the compounds **4b**_3_ (EC_50_ = 0.0330 ± 0.0009) and **4b**_4_ (EC_50_ = 0.0332 ± 0.0047) are approximately 650 time more active than sulfisoxazole (**1b**) (EC_50_ = 21.658 ± 0.0224). Their azetidinone analogues are 380 times (**5b**_3_, EC_50_ = 0.0574 ± 0.0025) and 560 times (**5b**_4_, EC_50_ = 0.0385 ± 0.0078) more active than sulfisoxazole.

#### 2.2.2.3. DPPH Radical Scavenging Assay

DPPH is a well-know radical which demonstrates a strong absorption band centered at about 517 nm, and it becomes colorless or pale yellow when it is neutralized. DPPH radical is scavenged by antioxidants through the donation of proton forming the reduced DPPH, and it is commonly used to evaluate the radical scavenging capacity of antioxidants [27]. The scavenging activities of the *N*-(arylidene)hydrazinoacetyl sulfonamides **4a**_1–6_, **4b**_1–6_ and *N*-(4-aryl-3-chloro-2-oxoazetidin-1-yl)aminoacetyl sulfonamides **5a**_1–6_, **5b**_1–6_, are presented in Table 7 and Table 8. All tested compounds are more active than their parent (sulfadiazine and sulfisoxazole) sulfonamides and some of them have a scavenging ability comparable with the scavenging ability of ascorbic acid. 

**Table 7:** DPPH radical scavenging ability of sulfadiazine derivatives **4a**_1–6_, **5a**_1–6_.

Sample	Scavenging ability (%)	Sample	Scavenging ability (%)
**4a**_1_	87.73 ± 0.69	**5a**_1_	67.18 ± 0.14
**4a**_2_	92.22 ± 0.89	**5a**_2_	78.25 ± 0.49
**4a**_3_	73.09 ± 0.50	**5a**_3_	80.94 ± 0.74
**4a**_4_	85.66 ± 0.89	**5a**_4_	71.52 ± 0.48
**4a**_5_	93.17 ± 0.64	**5a**_5_	61.28 ± 0.13
**4a**_6_	77.28 ± 0.83	**5a**_6_	71.61 ± 0.33
**1a**	11.15 ± 0.24	**AA**	97.08 ± 0.52

**Table 8:** DPPH radical scavenging ability of sulfisoxazole derivatives **4b**_1–6_, **5b**_1–6_.

Sample	Scavenging ability (%)	Sample	Scavenging ability (%)
**4b**_1_	48.17 ± 0.63	**5b**_1_	64.28 ± 0.49
**4b**_2_	6.43 ± 0.41	**5b**_2_	44.03 ± 0.13
**4b**_3_	67.39 ± 0.52	**5b**_3_	44.01 ± 0.86
**4b**_4_	42.95 ± 0.23	**5b**_4_	35.52 ± 0.48
**4b**_5_	61.20 ± 0.68	**5b**_5_	61.28 ± 0.13
**4b**_6_	82.65 ± 0.18	**5b**_6_	33.81 ± 0.09
**1b**	36.59 ± 0.08	**AA**	97.08 ± 0.52

The compounds obtained starting from sulfadiazine (compounds **4a**_1–6_, **5a**_1–6_, Table 7) are more active than sulfisoxazole derivatives **4b**_1–6_, **5b**_1–6_ (Table 8). In reference with sulfadiazine (1a), its *N*-(arylidene)hydrazinoacetyl derivatives **4a**_1–6_ are 6.5–8.4 times more active. Under similar conditions the azetidinone derivatives **5a**_1–6_ are slightly less active, being 5.5–7.3 more active than sulfadiazine. The most active compound is **4a**_5_ [*N^4^*-(4-hydroxybenzylidene)hydrazinoacetylamino-*N^1^*-(pyrimidin-2-yl)benzensulfonamide]; its scavenging ability (93.17 ± 0.64) being 8.4 time higher than sulfadiazine (11.15 ± 0.24) and comparable with ascorbic acid (97.08 ± 0.52).

## 3. Experimental

### 3.1. General Procedures

Melting points were measured using a Buchi Melting Point B-540 apparatus and are uncorrected. The FT-IR spectra were recorded on an ABB Bomen MB3000 spectrometer, over a 500–4000 cm^−1^ range, after 32 scans at a resolution of 4 cm^−1^. The spectra processing was carried out with Horizon MB^TM^ FTIR Software. The ^1^H-NMR (300 MHz) and ^13^C-NMR (75 MHz) spectra were obtained on a Bruker Avance ARX-300 spectrometer using tetramethylsilane as internal standard and DMSO-*d_6_* as solvent. The chemical shifts are shown in δ values (ppm). The progress of the reaction was monitored on TLC, using pre-coated Kieselgel 60 F254 plates (Merck) and the compounds were visualized by UV light.

### 3.2. Synthetic Procedures

#### 3.2.1. Preparation of *N*-(arylidene)hydrazinoacetyl Sulfonamides **4a**_1–6_; **4b**_1–6_

To a solution of hydrazinoacetyl sulfonamide derivatives (10 mmol) in ethanol 50% (200 mL), glacial acetic acid (0.5 mL) and the appropriate aldehyde (10 mmol) were added. The mixture was heated under reflux for 8 h, and then it was cooled at room temperature. The solid was filtered off, dried and recrystallized from isopropyl alcohol. 

*N^4^-(Benzylidene)hydrazinoacetylamino-N^1^-(pyrimidin-2-yl)benzensulfonamide* (**4a**_1_). Yield 87%, m.p. 200–202 °C; IR (KBr, cm^−1^): 3450 (-NH), 2853 (CH_2_-NH), 2924 (=CH- pyrimidine ring), 1693 (C=O), 1623 (HN-CO), 1591 (-C=C- aromatic ring), 1534 (N=CH), 1448 (-C=C- pyrimidine ring), 1313 (C-O), 1128 (-NH-SO_2_), 1097 (CH aromatic ring), 947 (S-N), 840 (S-C); ^1^H-NMR δ: 8.72–8.91 (dm, 3H, pyrimidine ring), 8.27 (s, 1H, CO-NH), 8.08 (s, 1H, N=CH), 7.15–7.90 (dm, 9H, *Ar*-H), 4.19 (s, 1H, -NH-SO_2_), 3.60 (d, 2H, CH_2_), 3.43 (m, 1H, HN-N).

*N^4^-(4-Fluorobenzylidene)hydrazinoacetylamino)-N^1^-(pyrimidin-2-yl*)benzensulfonamide (**4a**_2_). Yield 90%, m.p. 20–210 °C; IR (KBr, cm^−1^): 3350 (-NH), 2833 (CH_2_-NH), 2933 (=CH- pyrimidine ring), 1693 (C=O), 1622 (HN-CO), 1592 (-C=C- aromatic ring), 1535 (N=CH), 1455 (-C=C- pyrimidine ring), 1314 (C-O), 1229 (C-F), 1130 (-NH-SO_2_), 1099 (CH aromatic ring), 928 (S-N), 836 (S-C); ^1^H-NMR δ: 8.63-8.90 (dm, 3H, pyrimidine ring), 8.29 (s, 1H, CO-NH), 8.10 (s, 1H, N=CH), 7.1–7.95 (d, 8H, *Ar*-H), 4.20 (s, 1H, -NH-SO_2_), 3.79 (d, 2H, CH_2_), 3.45 (m, 1H, HN-N).

*N^4^-(4-Chlorobenzylidene)hydrazinoacetylamino-N^1^-(pyrimidin-2-yl)benzensulfonamide* (**4a**_3_). Yield 83% m.p. 20–207 °C; IR (KBr, cm^−1^): 3448 (-NH), 2830 (CH_2_-NH), 2930 (=CH- pyrimidine ring), 1695 (C=O), 1623 (HN-CO), 1591 (-C=C- aromatic ring), 1536 (N=CH), 1496 (-C=C- pyrimidine ring), 1312 (C-O), 1173 (-NH-SO_2_), 1084 (CH aromatic ring), 940 (S-N), 839 (S-C), 751 (C-Cl); ^1^H-NMR δ: 8.7–8.89 (dm, 3H, pyrimidine ring), 8.22 (s, 1H, CO-NH), 8.15 (s, 1H, N=CH), 7.0–7.76 (d, 8H, *Ar*-H), 4.17 (s, 1H, -NH-SO_2_), 3.61 (d, 2H, CH_2_), 3.40 (m, 1H, HN-N).

*N^4^-(4-Bromobenzylidene)hydrazinoacetylamino-N^1^-(pyrimidin-2-yl)benzensulfonamide* (**4a**_4_). Yield 81%, m.p. 212–213 °C; IR (KBr, cm^−1^): 3446 (-NH), 2850 (CH_2_-NH), 2940 (=CH- pyrimidine ring), 1690 (C=O), 1623 (HN-CO), 1591 (-C=C- aromatic ring), 1535 (N=CH), 1454 (-C=C- pyrimidine ring), 1314 (C-O), 1130 (-NH-SO_2_), 1079 (CH aromatic ring), 959 (S-N), 840 (S-C), 550 (C-Br); ^1^H-NMR δ: 8.71–8.89 (dm, 3H, pyrimidine ring), 8.21 (s, 1H, CO-NH), 8.18 (s, 1H, N=CH), 7.19–7.84 (d, 8H, *Ar*-H), 4.16 (s, 1H, -NH-SO_2_), 3.56 (d, 2H, CH_2_), 3.40 (m, 1H, HN-N).

*N^4^-(4-Hydroxybenzylidene)hydrazinoacetylamino-N^1^-(pyrimidin-2-yl)benzensulfonamide* (**4a**_5_). Yield 86%, m.p. 208–209 °C; IR (KBr, cm^−1^): 3505 (OH), 3335 (-NH), 2850 (CH_2_-NH), 2932 (=CH- pyrimidine ring), 1677 (C=O), 1622 (HN-CO), 1590 (-C=C- aromatic ring), 1539 (N=CH), 1514 (-C=C- pyrimidine ring), 1304 (C-O), 1168 (-NH-SO_2_), 1078 (CH aromatic ring), 965 (S-N), 839 (S-C); ^1^H-NMR δ: 8.56–8.89 (dm, 3H, pyrimidine ring), 8.24 (s, 1H, CO-NH), 8.07 (s, 1H, N=CH), 7.35–7.89 (d, 8H, *Ar*-H), 5.05 (s, 1H, Ar-OH), 4.17 (s, 1H, -NH-SO_2_), 3.63 (d, 2H, CH_2_), 3.44 (m, 1H, HN-N).

*N^4^-(4-Nitrobenzylidene)hydrazinoacetylamino-N^1^-(pyrimidin-2-yl)benzensulfonamide* (**4a**_6_). Yield 75%, m.p. 227–230 °C; IR (KBr, cm^−1^): 3445 (-NH), 2851 (CH_2_-NH), 2932 (=CH- pyrimidine ring), 1689 (C=O), 1623 (HN-CO), 1592 (-C=C- aromatic ring), 1536 (N=CH), 1514 (-C=C- pyrimidine ring), 1343 (C-NO_2_), 1316 (C-O), 1174 (-NH-SO_2_), 1078 (CH aromatic ring), 951 (S-N), 837 (S-C); ^1^H-NMR δ: 8.50–8.87 (dm, 3H, pyrimidine ring), 8.21 (s, 1H, CO-NH), 8.06 (s, 1H, N=CH), 7.33–7.90 (d, 8H, *Ar*-H), 4.16 (s, 1H, -NH-SO_2_), 3.70 (d, 2H, CH_2_), 3.41 (m, 1H, HN-N).

*N^4^-(Benzylidene)hydrazinoacetylamino-N^1^-(3,4-dimethyl-1,2-oxazol-5-yl)benzensulfonamide* (**4b**_1_). Yield 87%, m.p. 118–120 °C; IR (KBr, cm^−1^): 3465 (-NH), 2864 (CH_2_-NH), 1686 (C=O), 1625 (HN-CO), 1592 (-C=C- aromatic ring), 1518 (N=CH), 1496 (-C=C- oxazole ring), 1310 (C-O), 1151 (-NH-SO_2_), 1093 (CH aromatic ring), 956 (S-N), 830 (S-C); ^1^H-NMR δ: 8.41 (s, 1H, CO-NH), 8.12 (s, 1H, N=CH), 7.21–7.93 (dm, 9H, *Ar*-H), 4.62 (s, 1H, -NH-SO_2_), 3.59 (d, 2H, CH_2_), 3.24 (m, 1H, HN-N), 2.01–2.38 (s, 6H, 2CH_3_).

*N^4^-(4-Fluorobenzylidene)hydrazinoacetylamino-N^1^-(3,4-dimethyl-1,2-oxazol-5-yl)benzensulfonamide* (**4b**_2_). Yield 68%, m.p. 187–199 °C; IR (KBr, cm^−1^): 3452 (-NH), 2863 (CH_2_-NH), 1697 (C=O), 1631 (HN-CO), 1601 (-C=C- aromatic ring), 1507 (N=CH), 1482 (-C=C- oxazole ring), 1320 (C-O), 1225 (C-F), 1153 (-NH-SO_2_), 1097 (CH aromatic ring), 962 (S-N), 824 (S-C); ^1^H-NMR δ: 8.36 (s, 1H, CO-NH), 8.21 (s, 1H, N=CH), 7.18–7.91 (d, 8H, *Ar*-H), 4.65 (s, 1H, -NH-SO_2_), 3.62 (d, 2H, CH_2_), 3.38 (m, 1H, HN-N), 2.08–2.34 (s, 6H, 2CH_3_).

*N^4^-(4-Chlorobenzylidene)hydrazinoacetylamino-N^1^-(3,4-dimethyl-1,2-oxazol-5-yl)benzensulfonamide* (**4b**_3_). Yield 62%, m.p. 175–178 °C; IR (KBr, cm^−1^): 3451 (-NH), 2850 (CH_2_-NH), 1702 (C=O), 1622 (HN-CO), 1586 (-C=C- aromatic ring), 1540 (N=CH), 1483 (-C=C- oxazole ring), 1312 (C-O), 1169 (-NH-SO_2_), 1086 (CH aromatic ring), 957 (S-N), 830 (S-C), 814 (C-Cl); ^1^H-NMR δ: 8.31 (s, 1H, CO-NH), 8.16 (s, 1H, N=CH), 7.25–7.98 (d, 8H, *Ar*-H), 4.74 (s, 1H, -NH-SO_2_), 3.64 (d, 2H, CH_2_), 3.39 (m, 1H, HN-N), 2.08–2.40 (s, 6H, 2CH_3_).

*N^4^-(4-Bromobenzylidene)hydrazinoacetylamino-N^1^-(3,4-dimethyl-1,2-oxazol-5-yl)benzensulfonamide* (**4b**_4_). Yield 57%, m.p. 219 °C; IR (KBr, cm^−1^): 3439 (-NH), 2870 (CH_2_-NH), 1669 (C=O), 1625 (HN-CO), 1591 (-C=C- aromatic ring), 1522 (N=CH), 1481 (-C=C- oxazole ring), 1315 (C-O), 1156 (-NH-SO_2_), 1095 (CH aromatic ring), 940 (S-N), 832 (S-C), 555 (C-Br); ^1^H-NMR δ: 8.32 (s, 1H, CO-NH), 8.18 (s, 1H, N=CH), 7.36-7.94 (d, 8H, *Ar*-H), 4.61 (s, 1H, -NH-SO_2_), 3.65 (d, 2H, CH_2_), 3.39 (m, 1H, HN-N), 2.10–2.32 (s, 6H, 2CH_3_).

*N^4^-(4-Hydroxybenzylidene)hydrazinoacetylamino-N^1^-(3,4-dimethyl-1,2-oxazol-5-yl)benzensulfonamide* (**4b**_5_). Yield 63%, m.p. 190–192 °C; IR (KBr, cm^−1^): 3495 (C-OH), 3479 (-NH), 2859 (CH_2_-NH), 1659 (C=O), 1622 (HN-CO), 1593 (-C=C- aromatic ring), 1530 (N=CH), 1476 (-C=C- oxazole ring), 1323 (C-O), 1150 (-NH-SO_2_), 1094 (CH aromatic ring), 962 (S-N), 825 (S-C); ^1^H-NMR δ: 8.36 (s, 1H, CO-NH), 8.22 (s, 1H, N=CH), 7.31–7.84 (d, 8H, *Ar*-H), 4.92 (s, 1H, Ar-OH), 4.62 (s, 1H, -NH-SO_2_), 3.58 (d, 2H, CH_2_), 3.42 (m, 1H, HN-N), 2.08–2.32 (s, 6H, 2CH_3_).

*N^4^-(4-Nitrobenzylidene)hydrazinoacetylamino-N^1^-(3,4-dimethyl-1,2-oxazol-5-yl)benzensulfonamide* (**4b**_6_). Yield 71%, m.p. 280 °C; IR (KBr, cm^−1^): 3433 (-NH), 2842 (CH_2_-NH), 1683 (C=O), 1629 (HN-CO), 1593 (-C=C- aromatic ring), 1516 (N=CH), 1480 (-C=C- oxazole ring), 1342 (C-NO_2_), 1329 (C-O), 1161 (-NH-SO_2_), 1106 (CH aromatic ring), 951 (S-N), 838 (S-C); ^1^H-NMR δ: 8.28 (s, 1H, CO-NH), 8.21 (s, 1H, N=CH), 7.27–7.88 (d, 8H, *Ar*-H), 4.64 (s, 1H, -NH-SO_2_), 3.56 (d, 2H, CH_2_), 3.21 (m, 1H, HN-N), 2.11–2.34 (s, 6H, 2CH_3_).

#### 3.2.2. Preparation of *N*-(4-aryl-3-chloro-2-oxoazetidin-1-yl)aminoacetyl Sulfonamides **5a**_1–6_; **5b**_1–6_

To a solution of N-(arylidene)hydrazinoacetyl sulfonamides **4a**_1–6_; **4b**_1–6_ (2 mmol) in anhydrous 1,4-dioxane (50 mL), chloracetyl chloride (3 mmol) and triethylamine (2 mmol) were added dropwise at 0–5 °C. The mixture of reaction was stirred at room temperature for 3 h and the solid (triethylamine hydrochloride) was removed. The solution was heated under reflux for 5 h and then the solvent was evaporated under reduced pressure. The solid product was washed with water (20 mL), filtered off, dried and recrystallized from absolute ethanol. The progress of the reaction was monitored by silica gel coated TLC plates.

*N^4^-(2-Phenyl-3-chloro-4-oxoazetidin-1-yl)aminoacetylamino-N^1^-(pyrimidin-2-yl)benzensulfonamide* (**5a**_1_). Yield 75%, m.p. 218–220 °C; IR (KBr, cm^−1^): 3450 (-NH), 2869 (CH_2_-NH), 2945 (=CH- pyrimidine ring), 1745 (CO β-lactam), 1622 (HN-CO), 1591 (-C=C- aromatic ring), 1449 (-C=C- pyrimidine ring), 1314 (C-O), 1131 (-NH-SO_2_), 1078 (CH aromatic ring), 945 (S-N), 841 (S-C), 635 (C-Cl); ^1^H-NMR δ: 8.49–8.72 (dm, 3H, pyrimidine ring), 8.25 (s, 1H, CO-NH), 7.21–7.96 (dm, 9H, *Ar*-H), 5.45 (d, 1H, CH-*Ar*, azetidinone ring), 5.18 (d, 1H, CH-Cl), 4.27 (s, 1H, -NH-SO_2_), 3.75 (d, 2H, CH_2_), 3.39 (m, 1H, HN-N); ^13^C-NMR δ: 170.4 (CONH), 162.8 (CO, β-lactam), 126.7–140.9 (12 aromatic carbons), 118.9 (1C pyrimidine ring), 154.7 (3C pyrimidine ring), 67.8 (CH), 62.7 (CH-Cl), 57.7 (CH_2_); Anal. calcd for C_21_H_19_O_4_N_6_S: C 55.87, H 4.24, N 18.61; found: C 56.02, H 4.38, N 18.48.

*N^4^*-[2-(4-Fluoro)phenyl-3-chloro-4-oxoazetidin-1-yl]*aminoacetylamino-N^1^-(pyrimidin-2-yl)benzen-sulfonamide* (**5a**_2_). Yield 90%, m.p. 213–215 °C; IR (KBr, cm^−1^): 3355 (-NH), 2831 (CH_2_-NH), 2943 (=CH- pyrimidine ring), 1744 (CO β-lactam), 1624 (HN-CO), 1593 (-C=C- aromatic ring), 1508 (-C=C- pyrimidine ring), 1315 (C-O), 1230 (C-F), 1131 (-NH-SO_2_), 1100 (CH aromatic ring), 926 (S-N), 836 (S-C), 613 (C-Cl); ^1^H-NMR δ: 8.47–8.70 (dm, 3H, pyrimidine ring), 8.22 (s, 1H, CO-NH), 7.23–7.97 (d, 8H, *Ar*-H), 5.35 (d, 1H, CH-*Ar*, azetidinone ring), 5.04 (d, 1H, CH-Cl), 4.37 (s, 1H, -NH-SO_2_), 3.79 (d, 2H, CH_2_), 3.39 (m, 1H, HN-N); ^13^C-NMR δ: 169.2 (CONH), 162.5 (CO, β-lactam), 124.9–141.06 (12 aromatic carbons), 118.8 (1C pyrimidine ring), 151.8 (3C pyrimidine ring), 69.2 (CH), 61.04 (CH-Cl), 55.6 (CH_2_); Anal. calcd for C_21_H_18_O_4_N_6_SF: C 53.73, H 3.86, N 17.90; found: C 53.96, H 4.06, N 18.14.

*N^4^*-[2-(4-Chloro)phenyl-3-chloro-4-oxoazetidin-1-yl]*aminoacetylamino-N^1^-(pyrimidin-2-yl)benzen-sulfonamide* (**5a**_3_). Yield 85%, m.p. 201–203 °C; IR (KBr, cm^−1^): 3355 (-NH), 2830 (CH_2_-NH), 2944 (=CH- pyrimidine ring), 1745 (CO β-lactam), 1624 (HN-CO), 1592 (-C=C- aromatic ring), 1490 (-C=C- pyrimidine ring), 1314 (C-O), 1174 (-NH-SO_2_), 1088 (CH aromatic ring), 958 (S-N), 825 (S-C), 610 (C-Cl); ^1^H-NMR) δ: 8.48–8.74 (dm, 3H, pyrimidine ring), 8.24 (s, 1H, CO-NH), 7.19–7.96 (d, 8H, *Ar*-H), 5.38 (d, 1H, CH-*Ar*, azetidinone ring), 5.00 (d, 1H, CH-Cl), 4.4 (s, 1H, -NH-SO_2_), 3.75 (d, 2H, CH_2_), 3.39 (m, 1H, HN-N); ^13^C-NMR δ: 167.6 (CONH), 160.5 (CO, β-lactam), 126.7–142.6 (12 aromatic carbons), 120.5 (1C pyrimidine ring), 155.1 (3C pyrimidine ring), 68.7 (CH), 62.8 (CH-Cl), 54.4 (CH_2_); Anal. calcd for C_21_H_18_O_4_N_6_SCl: C 51.91, H 3.73, N 17.29; found: C 52.14, H 3.95, N 17.04.

*N^4^*-[2-(4-Bromo)phenyl-3-chloro-4-oxoazetidin-1-yl]*aminoacetylamino-N^1^-(pyrimidin-2-yl)benzen-sulfonamide* (**5a**_4_). Yield 88%, m.p. 206–208 °C; IR (KBr, cm^−1^): 3445 (-NH), 2848 (CH_2_-NH), 2943 (=CH- pyrimidine ring), 1739 (CO β-lactam), 1624 (HN-CO), 1590 (-C=C- aromatic ring), 1485 (-C=C- pyrimidine ring), 1315 (C-O), 1130 (-NH-SO_2_), 1067 (CH aromatic ring), 961 (S-N), 840 (S-C), 630 (C-Cl), 572 (C-Br); ^1^H-NMR δ: 8.47–8.71 (dm, 3H, pyrimidine ring), 8.22 (s, 1H, CO-NH), 7.16–7.85 (d, 8H, *Ar*-H), 5.36 (d, 1H, CH-*Ar*, azetidinone ring), 5.05 (d, 1H, CH-Cl), 4.27 (s, 1H, -NH-SO_2_), 3.79 (d, 2H, CH_2_), 3.39 (m, 1H, HN-N); ^13^C-NMR δ: 165.3 (CONH), 160.7 (CO, β-lactam), 126.6–136.6 (12 aromatic carbons), 118.7 (1C pyrimidine ring), 156.6 (3C pyrimidine ring), 68.2 (CH), 62.09 (CH-Cl), 57.5 (CH_2_); Anal. calcd for C_21_H_18_O_4_N_6_SBr: C 47.56, H 3.42, N 15.85; found: C 47.28, H 3.71, N 15.67.

*N^4^*-[2-(4-Hydroxy)phenyl-3-chloro-4-oxoazetidin-1-yl]*aminoacetylamino-N^1^-(pyrimidin-2-yl)benzen-sulfonamide* (**5a**_5_). Yield 80%, m.p. 231–233 °C; IR (KBr, cm^−1^): 3497 (C-OH), 3355 (-NH), 2843 (CH_2_-NH), 2943 (=CH- pyrimidine ring), 1740 (CO β-lactam), 1622 (HN-CO), 1590 (-C=C- aromatic ring), 1495 (-C=C- pyrimidine ring), 1315 (C-O), 1175 (-NH-SO_2_), 1067 (CH aromatic ring), 961 (S-N), 840 (S-C), 636 (C-Cl), ^1^H-NMR δ: 8.54–8.75 (dm, 3H, pyrimidine ring), 8.26 (s, 1H, CO-NH), 7.30–7.90 (d, 8H, *Ar*-H), 5.34 (d, 1H, CH-*Ar*, azetidinone ring), 5.06 (d, 1H, CH-Cl), 4.95 (s, 1H, Ar-OH), 4.25 (s, 1H, -NH-SO_2_), 3.64 (d, 2H, CH_2_), 3.40 (m, 1H, HN-N); ^13^C-NMR δ: 167.7 (CONH), 160.3 (CO, β-lactam), 114.7–132.1 (12 aromatic carbons), 119.5 (1C pyrimidine ring), 153.4 (3C pyrimidine ring), 72.9 (CH), 63.3 (CH-Cl), 54.2 (CH_2_); Anal. calcd for C_21_H_19_O_5_N_6_S: C 53.95, H 4.10, N 17.98; found: C 54.21, H 4.32, N 18.19.

*N^4^*-[2-(4-Nitro)phenyl-3-chloro-4-oxoazetidin-1-yl]*aminoacetylamino-N^1^-(pyrimidin-2-yl)benzen-sulfonamide* (**5a**_6_). Yield 86%, m.p. 200–203 °C; IR (KBr, cm^−1^): 3350 (-NH), 2846 (CH_2_-NH), 2942 (=CH- pyrimidine ring), 1743 (CO β-lactam), 1624 (HN-CO), 1593 (-C=C- aromatic ring), 1496 (-C=C- pyrimidine ring), 1343 (C-NO_2_), 1316 (C-O), 1131 (-NH-SO_2_), 1079 (CH aromatic ring), 951 (S-N), 839 (S-C), 635 (C-Cl); ^1^H-NMR) δ: 8.36–8.87 (dm, 3H, pyrimidine ring), 8.29 (s, 1H, CO-NH), 7.19–7.99 (d, 8H, *Ar*-H), 5.33 (d, 1H, CH-*Ar*, azetidinone ring), 5.02 (d, 1H, CH-Cl), 4.24 (s, 1H, -NH-SO_2_), 3.62 (d, 2H, CH_2_), 3.37 (m, 1H, HN-N); ^13^C-NMR δ: 168.3 (CONH), 161.9 (CO, β-lactam), 118.5–137.2 (12 aromatic carbons), 118.6 (1C pyrimidine ring), 155.8 (3C pyrimidine ring), 76.1 (CH), 64.3 (CH-Cl), 58.0 (CH_2_); Anal. calcd for C_21_H_18_O_6_N_7_S: C 50.80, H 3.65, N 19.75; found: C 50.64, H 3.89, N 19.97.

*N^4^-(2-Phenyl-3-chloro-4-oxoazetidin-1-yl)aminoacetylamino-N^1^-(3,4-dimethyl-1,2-oxazol-5-yl)benzen-sulfonamide* (**5b**_1_). Yield 51%, m.p. 212–213 °C; IR (KBr, cm^−1^): 3342 (-NH), 2860 (CH_2_-NH), 1743 (CO β-lactam), 1624 (HN-CO), 1591 (-C=C- aromatic ring), 1495 (-C=C- oxazole ring), 1318 (C-O), 1153 (-NH-SO_2_), 1092 (CH aromatic ring), 957 (S-N), 836 (S-C), 663 (C-Cl); ^1^H-NMR δ: 8.34 (s, 1H, CO-NH), 7.13–7.86 (dm, 9H, *Ar*-H), 5.37 (d, 1H, CH-*Ar*, azetidinone ring), 5.19 (d, 1H, CH-Cl), 4.57 (s, 1H, -NH-SO_2_), 3.51 (d, 2H, CH_2_), 3.11 (m, 1H, HN-N), 1.95–2.30 (s, 6H, 2CH_3_); ^13^C-NMR δ: 168.5 (CONH), 164.0 (CO, β-lactam), 151.4–155.8 (2C oxazole ring), 101.05 (1C oxazole ring), 118.8–135.3 (12 aromatic carbons), 77.2 (CH), 66.3 (CH-Cl), 59.2 (CH_2_), 8.48 (2CH_3_); Anal. calcd for C_22_H_22_O_5_N_5_S: C 56.40, H 4.73, N 14.95; found: C 56.63, H 4.94, N 15.18. 

*N^4^*-[2-(4-Fluorophenyl)-3-chloro-4-oxoazetidin-1-yl]*aminoacetylamino-N^1^-(3,4-dimethyl-1,2-oxazol-5-yl)benzensulfonamide* (**5b**_2_). Yield 76%, m.p. 209–211 °C; IR (KBr, cm^−1^): 3450 (-NH), 2861 (CH_2_-NH), 1739 (CO β-lactam), 1633 (HN-CO), 1591 (-C=C- aromatic), 1497 (-C=C- oxazole ring), 1322 (C-O), 1232 (C-F), 1153 (-NH-SO_2_), 1094 (CH aromatic ring), 961 (S-N), 836 (S-C), 662 (C-Cl); ^1^H-NMR δ: 8.24 (s, 1H, CO-NH), 7.10–7.85 (d, 8H, *Ar*-H), 5.38 (d, 1H, CH-*Ar*, azetidinone ring), 5.23 (d, 1H, CH-Cl), 4.57 (s, 1H, -NH-SO_2_), 3.57 (d, 2H, CH_2_), 3.41 (m, 1H, HN-N), 2.02–2.27 (s, 6H, 2CH_3_); ^13^C-NMR δ: 169.6 (CONH), 160.3 (CO, β-lactam), 118.6–139.9 (12 aromatic carbons), 99.56 (1C oxazole ring), 151.8–153.3 (2C oxazole ring), 75.9 (CH), 64.2 (CH-Cl), 56.2 (CH_2_), 10.84 (2CH_3_); Anal. calcd for C_22_H_21_O_5_N_5_SF: C 54.31, H 4.35, N 14.40; found: C 54.09, H 4.58, N 14.68. 

*N^4^*-[2-(4-Chlorophenyl)-3-chloro-4-oxoazetidin-1-yl]*aminoacetylamino-N^1^-(3,4-dimethyl-1,2-oxazol-5-yl)benzensulfonamide* (**5b**_3_). Yield 72%, m.p. 211–213 °C; IR (KBr, cm^−1^): 3361 (-NH), 2869 (CH_2_-NH), 1750 (CO β-lactam), 1620 (HN-CO), 1591 (-C=C- aromatic), 1495 (-C=C oxazole ring), 1318 (C-O), 1153 (-NH-SO_2_), 1092 (CH aromatic ring), 957 (S-N), 836 (S-C), 663 (C-Cl); ^1^H-NMR δ: 8.23 (s, 1H, CO-NH), 7.22–7.92 (d, 8H, *Ar*-H), 5.35 (d, 1H, CH-*Ar*, azetidinone ring), 5.20 (d, 1H, CH-Cl), 4.68 (s, 1H, -NH-SO_2_), 3.52 (d, 2H, CH_2_), 3.33 (m, 1H, HN-N), 2.05–2.36 (s, 6H, 2CH_3_); ^13^C-NMR (DMSO) δ in ppm: 170.3 (CONH), 163.6 (CO, β-lactam), 118.8–132.7 (12 aromatic carbons), 100.97 (1C oxazole ring), 150.4–152.7 (2C oxazole ring), 77.4 (CH), 66.3 (CH-Cl), 53.1 (CH_2_), 8.50 (2CH_3_); Anal. calcd for C_22_H_21_O_5_N_5_SCl: C 52.54, H 4.21, N 13.92; found: C 52.38, H 4.08, N 13.71.

*N^4^*-[2-(4-Bromophenyl)-3-chloro-4-oxoazetidin-1-yl]*aminoacetylamino-N^1^-(3,4-dimethyl-1,2-oxazol-5-yl)benzensulfonamide* (**5b**_4_). Yield 80%, m.p. 212–214 °C; IR (KBr, cm^−1^): 3386 (-NH), 2866 (CH_2_-NH), 1748 (CO β-lactam), 1626 (HN-CO), 1589 (-C=C- aromatic ring), 1483 (-C=C- oxazole ring), 1315 (C-O), 1158 (-NH-SO_2_), 1095 (CH aromatic ring), 960 (S-N), 859 (S-C), 660 (C-Cl), 556 (C-Br); ^1^H-NMR δ: 8.21 (s, 1H, CO-NH), 7.34–7.98 (d, 8H, *Ar*-H), 5.33 (d, 1H, CH-*Ar*, azetidinone ring), 5.19 (d, 1H, CH-Cl), 4.56 (s, 1H, -NH-SO_2_), 3.60 (d, 2H, CH_2_), 3.36 (m, 1H, HN-N), 2.08–2.28 (s, 6H, 2CH_3_); ^13^C-NMR δ: 171.3 (CONH), 160.7 (CO, β-lactam), 128.8–142.7 (12 aromatic carbons), 102.37 (1C oxazole ring), 151.7–153.5 (2C oxazole ring), 78.8 (CH), 67.0 (CH-Cl), 55.0 (CH_2_), 8.50 (2CH_3_); Anal. calcd for C_22_H_21_O_5_N_5_SBr: C 48.27, H 3.87, N 12.79; found: C 48.54, H 3.94, N 12.97.

*N^4^*-[2-(4-Hydroxyphenyl)-3-chloro-4-oxoazetidin-1-yl]*aminoacetylamino-N^1^-(3,4-dimethyl-1,2-oxazol-5-yl)benzensulfonamide* (**5b**_5_). Yield 55%, m.p. 210–212 °C; IR (KBr, cm^−1^): 3561 (C-OH), 3461 (-NH), 2855 (CH_2_-NH), 1746 (CO β-lactam), 1621 (HN-CO), 1590 (-C=C- aromatic), 1480 (-C=C- oxazole ring), 1308 (C-O), 1151 (-NH-SO_2_), 1093 (CH aromatic ring), 989 (S-N), 835 (S-C), 659 (C-Cl); ^1^H-NMR δ in ppm: 8.31 (s, 1H, CO-NH), 7.28–7.81 (d, 8H, *Ar*-H), 5.32 (d, 1H, CH-*Ar*, azetidinone ring), 5.10 (d, 1H CH-Cl), 4.84 (s, 1H, Ar-OH), 4.57 (s, 1H, -NH-SO_2_), 3.55 (d, 2H, CH_2_), 3.39 (m, 1H, HN-N), 2.04–2.28 (s, 6H, 2CH_3_); ^13^C-NMR δ: 175.2 (CONH), 168.2 (CO, β-lactam), 115.8–126.8 (12 aromatic carbons), 101.79 (1C oxazole ring), 148.9–150.5 (2C oxazole ring), 77.3 (CH), 64.8 (CH-Cl), 55.2 (CH_2_), 8.51 (2CH_3_); Anal. calcd for C_22_H_22_O_6_N_5_S: C 54.54, H 4.58, N 14.45; found: C 54.73, H 4.63, N 14.71. 

*N^4^*-[2-(4-Nitrophenyl)-3-chloro-4-oxoazetidin-1-yl]*aminoacetylamino-N^1^-(3,4-dimethyl-1,2-oxazol-5-yl)benzensulfonamide* (**5b**_6_). Yield 59%, m.p. 218–220 °C; IR (KBr, cm^−1^): 3415 (-NH), 2863 (CH_2_-NH), 1752 (CO β-lactam), 1624 (HN-CO), 1591 (-C=C- aromatic ring), 1490 (-C=C- oxazole ring), 1337 (C-O), 1329 (C-NO_2_) 1154 (-NH-SO_2_), 1092 (CH aromatic ring), 987 (S-N), 836 (S-C), 662 (C-Cl); ^1^H-NMR δ: 8.18 (s, 1H, CO-NH), 7.19–7.86 (d, 8H, *Ar*-H), 5.36 (d, 1H, CH-*Ar*, azetidinone ring), 5.20 (d, 1H, CH-Cl), 4.55 (s, 1H, -NH-SO_2_), 3.49 (d, 2H, CH_2_), 3.11 (m, 1H, HN-N); 2.09–2.27 (s, 6H, 2CH_3_); ^13^C-NMR δ: 170.7 (CONH), 165.1 (CO, β-lactam), 118.7–130.6 (12 aromatic carbons), 101.20 (1C oxazole ring), 148.8–150.4 (2C oxazole ring), 79.1 (CH), 65.4 (CH-Cl), 60.7 (CH_2_), 9.54 (2CH_3_); Anal. calcd for C_22_H_21_O_7_N_6_S: C 51.46, H 4.12, N 16.37; found: C 51.29, H 4.36, N 16.09. 

### 3.3. Biological Evaluation

#### 3.3.1. Antibacterial Assay

In order to evaluate the antibacterial activity of the synthesized compounds **4a**_1–6_, **4b**_1–6_, **5a**_1–6_ and **5b**_1–6_, a panel of three Gram positive (*Staphyloccoccus aureus* ATCC 6583, *Staphyloccoccus epidermidis* ATCC 12228, *Enterococcus faecalis* ATCC 25912) and six Gram negative (*Klebsiella pneumoniae* CIP 53153, *Proteus vulgaris* CIP 104989, *Citrobacter freundii* CIP 5732, *Enterobacter cloacae* CIP 103475, *Escherichia coli* ATCC 25922, *Pseudomonas aeruginosa* CIP 82118) bacterial strains were used. Minimum inhibitory concentrations (MICs) were assessed according to the guidelines of EUCAST Def. 3.1 [24]. Briefly, stock solutions were prepared by solving the substances mentioned above (200 mg) in dimethyl sulfoxide (DMSO, 19.5 mL). Using these solutions, series of two-fold dilutions were subsequently obtained. In a 9 cm diameter Petri dish, one milliliter of each dilution was mixed thoroughly with Mueller-Hinton agar (19 mL), sterilized by autoclaving and cooled to 50 °C. After this, the concentrations of the substances inside the medium were 512, 256, 128, 64, 32, 16, 8, 4, 2, and 1 μg/mL respectively. A blank plate (control of growth) was also prepared by mixing DMSO (1 mL) with molten agar (19 mL). From each bacterial strain, a 0.5 McFarland suspension was prepared in 0.85% saline solution and after that, the inoculum was standardized in order to assure 10^4^ colony-forming units (CFU) per spot (5 μL). All inoculated plates were incubated for 18 h at 36 °C. The MIC was interpreted as the lowest concentration of the substance that completely inhibits the growth of bacteria in the spot area. Each determination was performed in triplicate in order to accurately confirm the MIC values.

#### 3.3.2. Antioxidant Assays

The antioxidant activity was estimated using *in vitro* tests: ferric reducing power, total antioxidant capacity and radical scavenging ability. 

#### 3.3.2.1. Ferric Reducing Power

The ferric reducing power of the compounds was quantified by the method described by [25] with slight modifications. The sample solution (1 mL, 5 mg/mL in DMSO) was mixed with sodium phosphate buffer (1 mL, 0.2 M, pH 6.6) and potassium ferricyanide (1 mL, 1% w/v) in a test tube. The reaction mixture was incubated at 50 °C for 20 min in a water bath and then the reaction was stopped by adding trichloroacetic acid (1 mL, 10% w/v). After centrifugation of the mixture at 4500 rpm for 15 min, the upper layer of the solution (1mL) was collected and diluted further by adding deionised water (1 mL) and ferric chloride (0.2 mL, 0.1% w/v). After 5 min of incubation, the absorbance was measured at 700 nm against a blank (the mixture of DMSO with the reagents). A higher absorbance indicates a higher reducing power. For each sample it was calculated the effective concentration (EC_50_) and the reducing power was expressed in reference with ascorbic acid (AA) in the same concentration.

#### 3.3.2.2. Total Antioxidant Activity

The antioxidant activity of tested compounds was evaluated using the phosphomolybdenum method according to the procedure of [26] with minor modifications. The method is based on the reduction of Mo(VI) to Mo(V) by the tested compounds followed by the formation of a green phosphate/Mo(V) complex at acid pH. An aliquot of sample solution (50 µL, 5 mg/mL in DMSO) is mixed with the reagent solution (2 mL, 0.6 M sulphuric acid, 28 mM sodium phosphate and 4 mM ammonium molybdate). The samples were incubated at 95 °C for 90 min and then were cooled to room temperature. The absorbance was measured at 695 nm against a blank (DMSO mixed with reagent solution). For each sample the effective concentration (EC_50_) was calculated and the antioxidant activity was expressed in reference with ascorbic acid (**AA**) in the same concentration. 

#### 3.3.2.3. DPPH Radical Scavenging Assay

The radical scavenging activity of the tested compounds towards the radical 1,1-diphenyl-2-picrylhydrazyl (DPPH) was measured as described by [27] with slight modifications. The sample solution (50 µL, 20 mg/mL in DMSO) was mixed thoroughly with a solution of DPPH in methanol (2.95 mL, 0.1 mM). The sample was left for 30 min at room temperature, in the dark, and after that the absorbance was measured at 517 nm (A_s_). A methanol solution of DPPH was used as control sample (A_c_). The ability to scavenge the DPPH radical was calculated using the following formula:

% Inhibition = 100 × (Ac−As)/Ac

and it was expressed in reference with the radical scavenging activity of ascorbic acid (AA) in the same concentration. 

#### 3.3.3. Statistical Analysis

All assays (antimicrobial and antioxidant) were carried out in triplicate. Data were analysed by an analysis of variance (ANOVA) (*p* < 0.05) and were expressed as means ± SD. The total antioxidant antivity (EC_50_ values) were calculated by linear interpolation between values above and below 50% activity.

## 4. Conclusions

In this study new *N*-(arylidene)hydrazinoacetyl and new 2-azetidionone derivatives have been designed and synthesized starting from sulfadiazine and sulfizoxazole. The structures of all new compounds were proved using spectral methods. The compounds were evaluated for their antimicrobial and antioxidant activity. Although their antimicrobial potential was reduced, they shown excellent antioxidant properties; for some of them the potential is comparable with the antioxidant activity of ascorbic acid. These results support the antioxidant potential of the synthesized compounds and their applications in several disease mediated by reactive oxygen species (ROS) including the healing of the wounds.
